# Reversal of aging-related emotional memory deficits by norepinephrine via regulating the stability of surface AMPA receptors

**DOI:** 10.1111/acel.12282

**Published:** 2015-01-07

**Authors:** Yi Luo, Jun Zhou, Ming-Xing Li, Peng-Fei Wu, Zhuang-Li Hu, Lan Ni, You Jin, Jian-Guo Chen, Fang Wang

**Affiliations:** 1 Department of Pharmacology, School of Basic Medicine, Tongji Medical College, Huazhong University of Science and TechnologyWuhan, 430030, China; 2 Key Laboratory of Neurological Diseases (HUST), Ministry of Education of ChinaWuhan, 430030, China; 3 The Key Laboratory for Drug Target Researches and Pharmacodynamic Evaluation of Hubei ProvinceWuhan, 430030, China; 4 The Institute of Brain Research, Huazhong University of Science and TechnologyWuhan, 430030, China

**Keywords:** aging, AMPA receptor, desipramine, emotional memory, long-term potentiation, norepinephrine

## Abstract

Aging-related emotional memory deficit is a well-known complication in Alzheimer's disease and normal aging. However, little is known about its molecular mechanism. To address this issue, we examined the role of norepinephrine (NE) and its relevant drug desipramine in the regulation of hippocampal long-term potentiation (LTP), surface expression of AMPA receptor, and associative fear memory in rats. We found that there was a defective regulation of NE content and AMPA receptor trafficking during fear conditioning, which were accompanied by impaired emotional memory and LTP in aged rats. Furthermore, we also found that the exogenous upregulation of NE ameliorated the impairment of LTP and emotional memory via enhancing AMPA receptor trafficking in aged rats, and the downregulation of NE impaired LTP in adult rats. Finally, acute treatment with NE or desipramine rescued the impaired emotional memory in aged rats. These results imply a pivotal role for NE in synaptic plasticity and associative fear memory in aging rats and suggest that desipramine is a potential candidate for treating aging-related emotional memory deficit.

## Introduction

Abundant evidence indicates that past emotional event is typically better remembered than neutral event in healthy young animals and humans (Cahill & McGaugh, 1995; Bass *et al*., 2013; McGaugh, 2013). However, the enhancement of emotional memory appears to be at least partially impaired in older humans (Kensinger *et al*., 2002). A variety of learning paradigms have been used for evaluating emotional memory, and among them, contextual fear conditioning is one of the most frequently used and well-recognized animal models. Old rodents have been reported to display decreased ability of learning and memory in contextual fear conditioning (Bergado *et al*., 2011; Neff *et al*., 2013), but the molecular mechanism underlying this phenomenon has not been clarified.

Noradrenergic activation that occurs during or immediately after an emotional event critically participates in modulating emotional memory in healthy young animals and humans (Cahill & McGaugh, 1995; Hu *et al*., 2007). During emotional event, norepinephrine (NE) is mainly released from neurons originating in the locus coeruleus (LC). These neurons project widely to several brain regions, including the hippocampus and amygdala, the key regions involved in the encoding and expression of emotional memory (Hu *et al*., 2007; Bass *et al*., 2013; Opmeer *et al*., 2013; Soya *et al*., 2013). NE activates adenylyl cyclase and cAMP-dependent protein kinase (PKA) and calcium/calmodulin-dependent protein kinase II (CaMKII) via β-adrenergic receptors (β-AR), then facilitates middle frequency stimulation (MFS)-induced long-term potentiation (LTP) in the hippocampus of young rodents (Katsuki *et al*., 1997; Hu *et al*., 2007). On the other side, degenerative processes of LC have been described in patients with mild cognitive impairment or early stage of Alzheimer's disease (AD; Grudzien *et al*., 2007), supporting a speculation that the deficiency of NE might mediate the aging-related emotional memory deficit, even though the underlying mechanism remains to be elucidated.

It has been reported that the trafficking of glutamate receptor 1 (GluR1) subunit-containing AMPA receptor (AMPAR) into synapse contributes considerably to the synaptic strengthening during LTP induction (Citri & Malenka, 2008). Phosphorylation of Ser831 and Ser845 sites of GluR1 is essential for the trafficking and function of AMPAR, which is the foundation of synaptic plasticity and learning and memory (Hu *et al*., 2007). Cognitive decline during normal aging does not seem to be characterized by dramatic neuronal cell death (Burke & Barnes, 2006). Otherwise, it appears to be mediated through alterations in synaptic glutamate receptors such as AMPAR (Henley & Wilkinson, 2013). To our knowledge, no studies have directly assessed the trafficking of AMPAR in animal models of normal aging. Our previous study found that the protein level of total GluR1 had no significant change in the hippocampus of aged rat (Yu *et al*., 2011); however, the trafficking of GluR1 in old individuals during emotional event needs to be investigated further.

Long-term potentiation, one of the prime candidates for mediating learning and memory, is widely believed to be a cellular substrate of memory process. The successful vs. unsuccessful induction of LTP can serve as a ‘diagnostic’ measure with which to assess the functional state of a brain structure (Segev *et al*., 2013). Aged animals show either a higher threshold for LTP induction or a decreased level of LTP induction compared with young animals (Henley & Wilkinson, 2013). Our previous study showed that aging-induced LTP impairment was reversed by reductants via regulation of thiol redox and *N*-methyl-d-aspartic acid receptor (NMDAR) function in hippocampal slices (Yang *et al*., 2010). Thus, it deserves further study to investigate whether enhancing the function of AMPAR is also capable of reversing the impairment of LTP and rescuing aging-related deficit of emotional memory in normally aged animals.

Desipramine is a classic antidepressant that selectively inhibits NE reuptake and affects the noradrenergic system (Chamberlain & Robbins, 2013). Desipramine improves mood by raising the level of NE in CNS (Dell'Osso *et al*., 2011), but the effect of desipramine on aging-related emotional memory deficit has not been examined.

In this study, we firstly testified whether aging-related emotional memory deficit was accompanied by dysregulation of AMPAR and NE level in the hippocampus. Secondly, we determined whether enhancing NE level was capable of promoting the surface expression of AMPAR to rescue the impaired LTP in aged rats. Thirdly, we further investigated the underlying molecular mechanism in animal models of normal aging. Fourthly, we asked whether dysregulation of NE in adult rats is able to mimic the impaired LTP in aged rats. Finally, we examined the effect of NE and desipramine on aging-related emotional memory deficit.

## Results

### The learning ability in contextual fear conditioning and hippocampal LTP is impaired in aged rats

**Figure 1:**
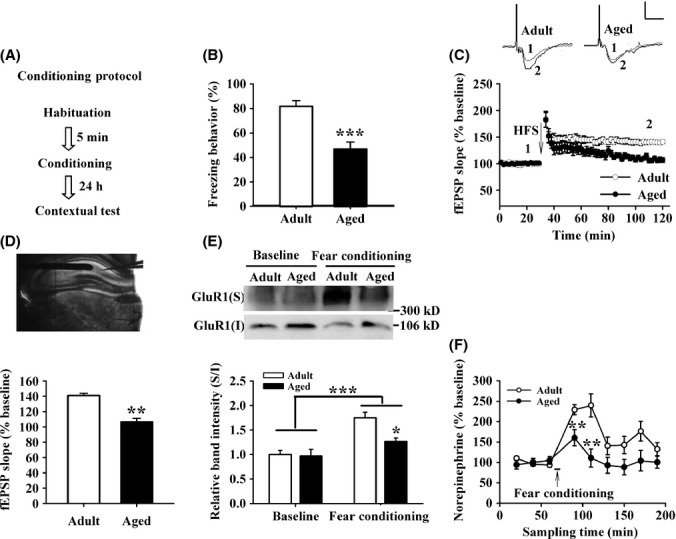
Learning ability in contextual fear conditioning and hippocampal long-term potentiation (LTP) is impaired in aged rats, accompanied by the decrease in norepinephrine (NE) level and surface expression of GluR1. (A) The contextual fear conditioning procedure. (B) The freezing behavior was significantly reduced in aged rats (*n* = 7) 24 h after conditioning training when compared with adult rats (*n* = 10). (C) Time course of the field excitatory postsynaptic potential (fEPSP) evoked by stimulation of Schaffer collateral (SC) synapse was recorded in CA1 slices from adult (*n* = 12 slices from seven rats) and aged rats (*n* = 10 slices from six rats). Calibration: 1 mV, 10 ms. (D) The histogram showing the level of LTP at 90 min after high-frequency stimulation (three trains at 100 Hz for 1 s with 30 s interval between trains) in the SC-CA1 pathway in adult and aged rats. The upper: the location of recording (right) and stimulating (left) electrodes in the SC-CA1 pathway of hippocampal slice. (E) The surface expression of GluR1 was decreased in aged rats (*n* = 6) at 1 h after fear conditioning compared with adult rats (*n* = 6). (F) The extracellular NE concentration was decreased in aged rats (*n* = 7) at 20 and 40 min after fear conditioning when compared with adult rats (*n* = 8). *P < 0.05, **P < 0.01, ***P < 0.001 vs. adult.

Firstly, we examined the learning and memory of rats in contextual fear conditioning (Fig.1A). Although aged rats exhibited more immobility time than adult rats before training, there were no significant changes in the percentage of freezing behavior (*F*_1,15_ = 3.475, *P* = 0.082, NS; one-factor ANOVA with age as the factor; Fig. S1A). The freezing behavior of both adult and aged rats was increased during the training of fear conditioning without any significant difference between the two groups (*F* = 0.265, NS; Fig. S1B; two-factor repeated measures ANOVA with the point of fear conditioning training as the within-subjects factor and age as the between-subjects factor), indicating that aged rats exhibited normal acquisition of fear memory. However, aged rats exhibited reduced freezing behavior 24 h after training compared with adult rats (adult: 81.74 + 4.51%, aged: 46.84 + 5.61%; *F*_1,15_ = 23.89, *P* < 0.001; one-factor ANOVA with age as the factor; Fig.1B), suggesting that aging impairs the consolidation of contextual fear memory. Moreover, aged rats showed a higher activity suppression ratio when compared with adult rats (adult: 0.15 + 0.03, aged: 0.36 + 0.02; *F*_1,15_ = 25.12, *P* < 0.001; one-factor ANOVA with age as the factor; Fig. S1C).

To exclude the possibility that nociception was altered with aging, the pain thresholds of adult and aged rats to the electric foot shock were examined. There was no difference between adult and aged rats in flinching (*F*_1,16_ = 2.138, NS; one-factor ANOVA with age as the factor) and jumping (*F*_1,15_ = 1.220, NS; one-factor ANOVA with age as the factor) in response to gradually increased intensity of electric shock (Fig. S1D and E), indicating that the impairment of fear memory is not due to pain sensitivity in aged rats.

To assess the possible general behavioral defects in aging, we performed a 30-min analysis of locomotive and anxiety-related behavior in the open-field test. It was found that there were no differences in the locomotive activity (*F*_1,18_ = 1.946, NS; one-factor ANOVA with age as the factor; Fig. S1F) and the percentage of time spent in the center (*F*_1,18_ = 1.255, NS; one-factor ANOVA with age as the factor) (Fig. S1G). These results suggest that there are no changes in the level of innate fear and anxiety in aged rats, and the observed differences in fear conditioning are not likely attributed to a sensory or motor deficit in aged rats. We also measured the short-term memory 2 h after training (Fig. S3A). As shown in Fig. S3B, aged rats exhibited reduced freezing behavior when compared to adult rats (adult: 86.68 + 2.80%, aged: 59.96 + 9.37%; *F*_1,17_ = 8.19, *P* = 0.011; one-factor ANOVA with age as the factor).

We next evaluated high-frequency stimulation (HFS)-induced LTP in the Schaffer collateral (SC)-CA1 pathway in brain slices from adult and aged rats. At 90 min after LTP induction, the slope of field excitatory postsynaptic potentials (fEPSPs) was still potentiated in adult rats, but returned to near baseline values in aged rats (adult: 140.94 + 2.88%, aged: 106.63 + 4.49%; *F*_1,20_ = 43.589, *P* < 0.001; one-factor ANOVA with age as the factor; Fig.1C,D). We also carried out control experiments to show that the field responses were stable at baseline in the SC-CA1 pathway in adult and aged rats (Fig. S2). These results suggest that aged rats show a remarkable impairment of LTP in the SC-CA1 pathway when compared with adult rats.

### The surface expression of GluR1 and hippocampal NE levels is reduced during contextual fear conditioning in aged rats

To assess AMPAR trafficking during emotional event, we next performed cross-linking assay of surface receptor to investigate the response of GluR1 to fear conditioning. Two-factor ANOVA analysis with age as the within-subjects factor and the fear conditioning as the between-subjects factor showed that aging had a significant effect on the surface expression of GluR1 (*F* = 6.072, *P* = 0.018), and fear conditioning significantly increased the surface expression of GluR1 (*F* = 27.596, *P* < 0.001). Moreover, the interaction of age and fear conditioning also affected the surface expression of GluR1 (*F* = 5.189, *P* = 0.034; Fig.1E). On the contrary, age (*F* = 0.105, *P* = 0.75) or fear conditioning (*F* = 0.071, *P* = 0.793) or the interaction of them (*F* = 0.333, *P* = 0.57) had no effect on GluR2 expression in the surface pool (Fig. S3D).

Considering that NE levels in several brain regions are altered during emotional events and GluR1 trafficking is modulated by NE (Hu *et al*., 2007), we wondered whether extracellular NE concentration in the hippocampus of aged and adult rats underwent alterations during fear conditioning. As shown in Fig.1F, two-factor repeated measure ANOVA with the time of measure as the within-subjects factor and age as the between-subjects factor showed that the extracellular concentration of NE in hippocampal CA1 region of aged and adult rats increased significantly after fear conditioning (*F* = 11.179, *P* = 0.004). The interaction of age and fear conditioning also affected the extracellular concentration of NE (*F* = 5.817, *P* = 0.023). However, the extracellular concentration of NE in aged rats was not increased as significantly as that in adult rats at 20 min (adult: 229.17 + 12.22%, aged: 160.45 + 19.79%; *F* = 9.240, *P* = 0.009) and 40 min (adult: 239.71 + 28.15%, aged: 111.00 + 22.17%; *F* = 12.367, *P* = 0.004) after fear conditioning. The level of basal extracellular NE in hippocampal CA1 region of aged rats was also significantly lower than that of adult rats (adult: 751.96 + 59.15 pm, aged: 160.69 + 56.27 pm;*F*_1,12_ = 52.558, *P* < 0.001; one-factor ANOVA with age as the factor; Fig. S3E). Previous study has reported that systemic administration of epinephrine elevates NE level in CNS (Chen & Williams, 2012). As shown in Fig. S3F, two-factor repeated measure ANOVA with the time of measure as the within-subjects factor and age as the between-subjects factor showed that the level of NE was increased after administration of epinephrine (*F* = 10.263, *P* = 0.02), but the interaction of age and epinephrine had no significant effect (*F* = 3.457, *P* = 0.123). Moreover, the levels of NE was lower in aged rats than that in adult rats at 20 min (adult: 303.21 + 18.80%, aged: 148.08 + 31.38%; *F* = 19.266, *P* = 0.001) and 40 min (adult: 244.83 + 35.29%, aged: 114.89 + 25.94%; *F* = 8.275, *P* = 0.015) after epinephrine (0.5 mg kg^−1^) administration.

### NE treatment increases the amplitude of hippocampal LTP in aged rats

**Figure 2:**
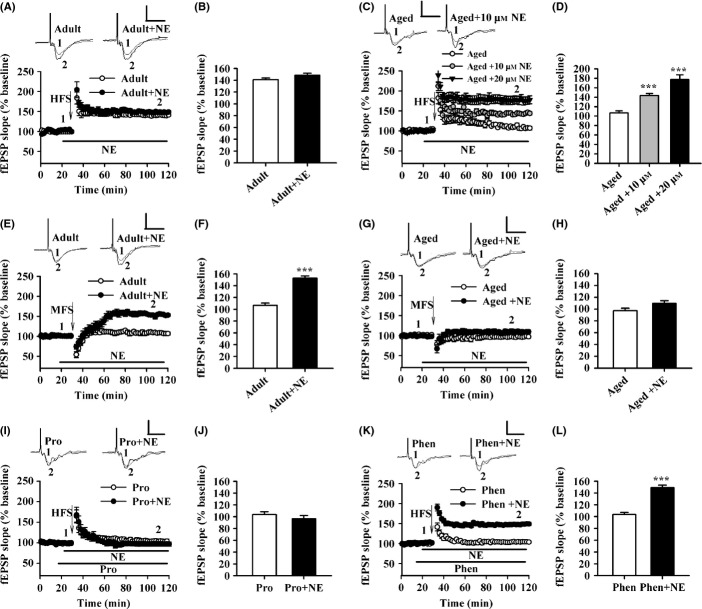
Norepinephrine (NE) increases the amplitude of long-term potentiation (LTP) in aged rats, but without effects on the threshold for LTP induction. (A) Time course of the field excitatory postsynaptic potential (fEPSP) from adult (*n *= 12 slices from seven rats) and NE-treated adult rats (*n* = 7 slices from six rats). (B) The histogram showing the level of LTP at 90 min after high-frequency stimulation (HFS) in adult and NE-treated adult rats. (C) Time course of the fEPSP from the slices of aged (*n* = 10 slices from six rats) and 10 μmNE-treated (*n* = 11 slices from seven rats) and 20 μmNE-treated aged rats (*n* = 9 slices from six rats). (D) The histogram showing the level of LTP at 90 min after HFS in aged and 10 μmNE and 20 μmNE-treated aged rats. ***P < 0.001 vs. aged. (E) Time course of the fEPSP from adult (*n* = 10 slices from six rats) and NE-treated adult (*n* = 8 slices from six rats). The middle frequency stimulation: MFS 900 trains at 10 Hz for 90 s. (F) The histogram showing the level of LTP at 90 min after MFS in adult and NE-treated adult rats. ***P < 0.001 vs. adult. (G) Time course of the fEPSP from aged (*n* = 10 slices from six rats) and NE-treated aged rats (*n* = 7 slices from four rats). (H) The histogram showing the level of LTP at 90 min after MFS in adult and NE-treated aged rats. (I) Time course of the fEPSP from 10 μm propranolol (Pro)-treated aged (*n* = 9 slices from five rats) and 10 μm Pro + 10 μmNE-treated aged (*n* = 9 slices from five rats) rats. (J) The histogram showing the level of LTP at 90 min after HFS in the two groups. (K) Time course of the fEPSP from 50 μm phentolamine (Phen)-treated aged (*n* = 9 slices from five rats) and 50 μm Phen + 10 μmNE-treated aged (*n* = 9 slices from five rats) rats. (L) The histogram showing the level of LTP at 90 min after HFS in the two groups. ***P < 0.001 vs. Phen-treated aged rats. Calibration: 1 mV, 10 ms.

As NE was insufficient during fear conditioning and hippocampal LTP was impaired in aged rats, we next examined whether NE treatment can rescue aging-related deficits. Bath application of NE (10 or 20 μm) for 10 min significantly increased the magnitude of LTP in aged rats (aged: 106.63 + 4.49%, aged + 10 μm NE: 143.76 + 4.03%, aged + 20 μm NE: 177.53 + 9.91%; *F*_2,27_ = 30.247, *P* < 0.001; one-factor ANOVA with the treatment as the factor; Fig.2C,D). Consistent with previous reports (Katsuki *et al*., 1997; Hu *et al*., 2007), bath application of NE had no effect on LTP of adult rats (*F*_1,17_ = 2.660, NS; one-factor ANOVA with the treatment as the factor; Fig.2A,B). Furthermore, application of NE (10 μm) for 10 min showed a lower threshold for LTP induction in the hippocampal slices of adult rats (adult: 106.57 + 3.86%, adult + NE: 152.78 + 3.57%, *F*_1,16_ = 73.885, *P* < 0.001; one-factor ANOVA with the treatment as the factor; Fig.2E,F), but not aged rats (*F*_1,15_ = 3.488, NS; one-factor ANOVA with the treatment as the factor; Fig.2G,H). Taken together, these results suggest that NE increases the amplitude of LTP, without affecting the threshold of induction in the hippocampal slices of aged rats.

### NE treatment rescues LTP impairment via facilitating surface delivery of GluR1 in aged rats

To further characterize the signaling events that lead to the rescue of LTP by NE, different pharmacological reagents were applied. As shown in Fig.2, after pretreated with reagents for 5 min, the effects of NE (10 μm) for 10 min on hippocampal LTP was observed. It was found that propranolol (Pro, 10 μm), a selective blocker of β-adrenoceptor (β-AR), completely blocked NE-induced increase in the level of LTP in hippocampal slices of aged rats (Pro: 103.65 + 4.61%, Pro + NE: 96.78 + 5.11%; *F*_1,16_ = 0.946, NS; one-factor ANOVA with the treatment as the factor; Fig.2I,J), while phentolamine (Phen, 50 μm), a selective blocker of α-adrenoceptor (α-AR), had no effect (Phen: 103.75 + 3.26%, Phen + NE: 149.02 + 4.32%; *F*_1,16_ = 69.791, *P* < 0.001; one-factor ANOVA with the treatment as the factor; Fig.2K,L). These results suggest that NE increases the amplitude of LTP via β-AR, but not α-AR in the hippocampal slices of aged rats.

**Figure 3:**
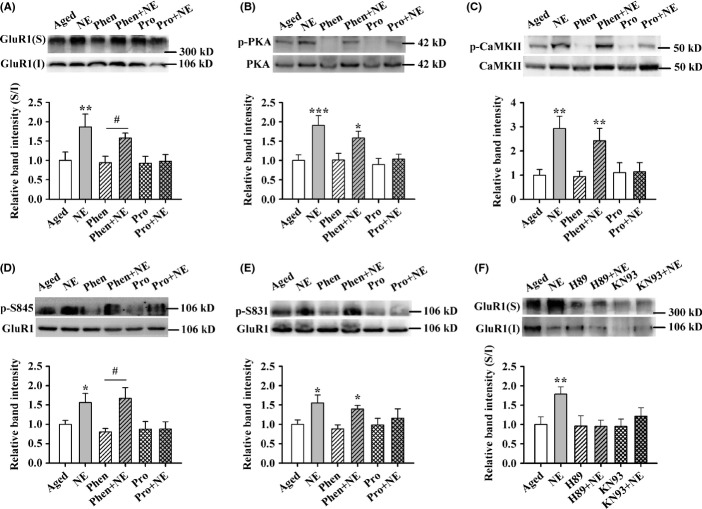
Norepinephrine (NE) rescues long-term potentiation (LTP) impairment via facilitating the surface delivery of GluR1 in the hippocampus of aged rats. (A) The histogram showing the surface expression of GluR1 was enhanced by treated with 10 μmNE for 10 min, and this response was prevented by pretreatment with 10 μm Pro, but not 50 μm Phen for 5 min (*n* = 6). **P < 0.01 vs. aged rats, ^#^*P* < 0.05 vs. Phen-treated aged rats. (B) The histogram showing that the phosphorylation level of cAMP-dependent protein kinase (PKA) was enhanced by NE, and this effect was inhibited by pretreatment with Pro, but not Phen (*n* = 7). *P < 0.05, ***P < 0.001 vs. aged rats. (C) The histogram showing that the phosphorylation level of calcium/calmodulin-dependent protein kinase II (CaMKII) was enhanced by NE, which was attenuated by pretreatment with Pro, but not Phen (*n* = 6). **P < 0.01 vs. aged rats. (D) The histogram showing that the phosphorylation level of GluR1-845 was enhanced by NE, which was inhibited by pretreatment with Pro, but not Phen (*n* = 6). *P < 0.05 vs. aged, ^#^*P* < 0.05 vs. Phen-treated aged rats. (E) The histogram showing that the phosphorylation level of GluR1-831 was enhanced by NE and abolished by pretreatment with Pro but not Phen (*n* = 6). *P < 0.05 vs. aged. (F) The histogram showing that the surface expression of GluR1 was enhanced by treatment with 10 μmNE for 10 min and abolished by pretreatment with 10 μmH89 or 20 μmKN93 for 5 min (*n* = 6). **P < 0.01 vs. aged rats.

Next, as shown in Fig.3, GluR1 in the surface pool of aged rats was obviously increased after NE treatment (aged: 1.00 + 0.19, aged + NE: 1.92 + 0.14). This effect was prevented by pretreatment with Pro but not Phen (*F*_5,30_ = 3.885, *P* = 0.008; one-factor ANOVA with the treatment as the factor; Fig.3A). Consistent with a previous study that β-AR activation couples to PKA and CaMKII phosphorylation in young rodents (Hu *et al*., 2007), we found that NE increased the phosphorylation levels of PKA (aged: 1.00 + 0.16, aged + NE: 1.95 + 0.35) and CaMKII (aged: 1.00 + 0.28, aged + NE: 3.29 + 0.40), and the effects were inhibited by pretreatment with Pro, but not Phen in aged rats (PKA, *F*_5,36_ = 6.409, *P* < 0.001; CaMKII, *F*_5,30_ = 5.851, *P* = 0.001; one-factor ANOVA with the treatment as the factor; Fig.3B,C). The GluR1 subunit contains two key phosphorylation sites, Ser845 (PKA site) and Ser831 (PKC/CaMKII site) in its carboxyl terminus, which regulate GluR1 trafficking and function *in vitro* and *in vivo* (Banke *et al*., 2000; Hu *et al*., 2007). As shown in Fig.3D,E, the phosphorylation levels of GluR1-845 (aged: 1.00 + 0.12, aged + NE: 1.44 + 0.12) and GluR1-831 (aged: 1.00 + 0.08, aged + NE: 2.05 + 0.35) were increased by NE treatment, and the effects could be blocked by pretreatment with Pro but not Phen in aged rats (Ser845, *F*_5,30_ = 2.605, *P* = 0.045; one-factor ANOVA with the treatment as the factor; Ser831, *F*_5,30_ = 4.119, *P* = 0.006; one-factor ANOVA with the treatment as the factor). In addition, we found that the total expression of GluR1 (*F*_5,30_ = 0.356, NS; one-factor ANOVA with the treatment as the factor) was not significantly affected by NE (Fig. S4A). These results suggest that the targets for NE may be Ser845 and Ser831 sites of GluR1 in aged rats.

Furthermore, both PKA inhibitor H89 (10 μm) and CaMKII inhibitor KN93 (20 μm) attenuated NE-induced increase in surface expression of GluR1 (*F*_5,30_ = 3.155, *P* = 0.021; one-factor ANOVA with the treatment as the factor; Fig.3F). We also found that the phosphorylation level of PKC (*F*_5,30_ = 0.268, NS) and ERK (*F*_5,30_ = 0.657, NS; one-factor ANOVA with the treatment as the factor) was not significantly affected by NE (Fig. S4B,C). However, the phosphorylation level of CREB was increased by NE, and this effect was largely weakened by pretreatment with Pro (*F*_5,30_ = 5.975, *P* = 0.001; one-factor ANOVA with the treatment as the factor) or H89 (10 μm;*F*_5,30_ = 9.186, *P* < 0.001; one-factor ANOVA with the treatment as the factor), but not Phen in aged rats (Fig. S4D,E). These results suggest that NE increases the surface expression of GluR1 via PKA and CaMKII pathways in aged rats.

**Figure 4:**
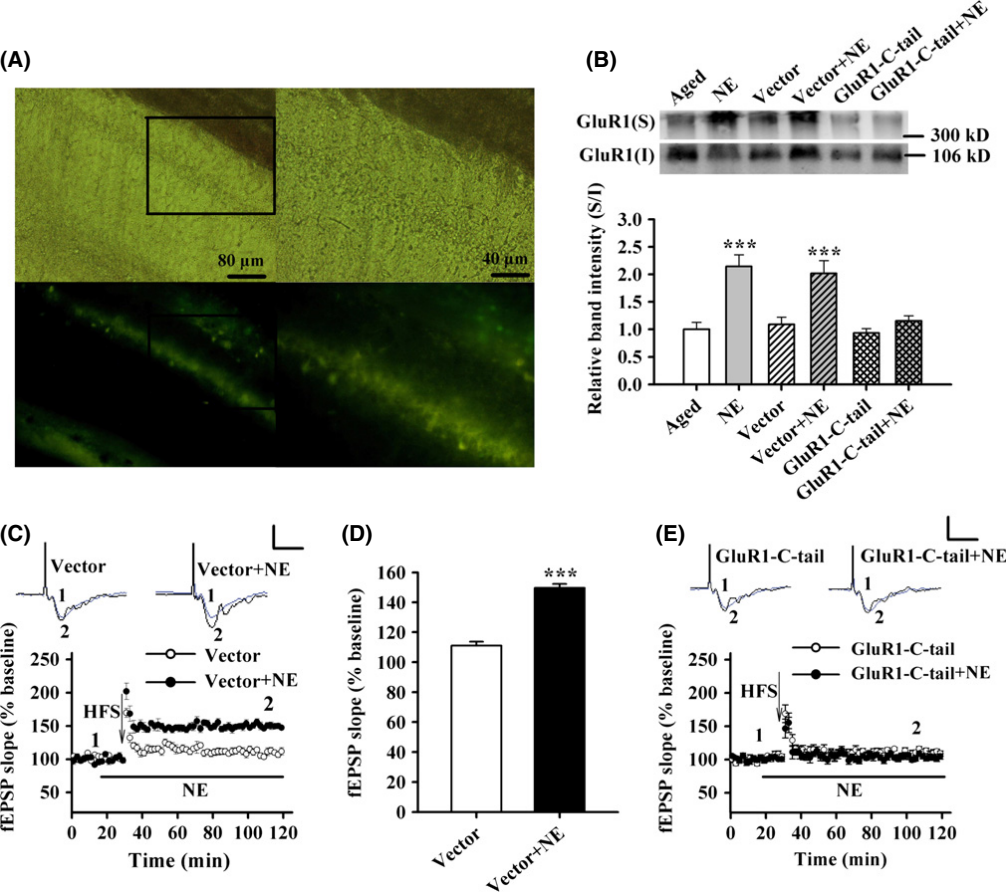
Overexpression of GluR1-C-terminus abolishes the effect of norepinephrine (NE) on long-term potentiation (LTP) in aged rats. (A) Representative images of transmitted light (upper) and fluorescence (lower) of a coronal section of the left CA1 at 36 h after injection of adenovirus. Fluorescence (lower) indicated the successful infection of adenovirus. The corresponding enlarged images of the left were shown on the right. (B) The histogram showing that the surface expression of GluR1 was enhanced by treatment with 10 μmNE for 10 min, and this effect was prevented by overexpression of GluR1 carboxyl terminus (*n* = 8), but not control adenoviral vectors. ***P < 0.001 vs. aged rats. (C) Time course of the field excitatory postsynaptic potential (fEPSP) from aged rats (*n* = 9 slices from five rats) and 10 μmNE-treated aged rats with control vector infection (*n* = 9 slices from five rats). (D) The histogram showing the level of LTP at 90 min after high-frequency stimulation in the two groups. ***P < 0.001 vs. aged rats with control vector infection. (E) Time course of the fEPSP from aged rats (*n* = 9 slices from five rats) and 10 μmNE-treated aged rats (*n* = 8 slices from five rats) with overexpression of GluR1 carboxyl terminus. Calibration: 1 mV, 10 ms.

To further confirm that NE facilitates the surface expression of GluR1 via increasing phosphorylation of Ser845 and Ser831 sites in aged rat hippocampus, the aged rats were bilaterally injected with GluR1-C-tail overexpressing or vector adenovirus into hippocampal CA1 region, which contains Ser845 and Ser831 sites. (Fig.4A). Thirty-six hours after injection, the increased surface expression of GluR1 induced by NE was largely prevented by GluR1-C-tail overexpression (1.24 + 0.16), but not vector adenovirus (1.86 + 0.27; *F*_5,42_ = 11.792, *P* < 0.001; one-factor ANOVA with the adenovirus as the factor; Fig.4B). Moreover, NE-facilitated LTP was also inhibited by GluR1-C-tail overexpression (*F*_1,15_ = 0.349, NS; one-factor ANOVA with the adenovirus as the factor; Fig.4E), but not affected by vector adenovirus (*F*_1,16_ = 66.224, *P* < 0.001; one-factor ANOVA with the vector adenovirus as the factor; Fig.4C,D). Taken together, these results suggest that NE rescues LTP impairment via facilitating surface delivery of GluR1 in aged rats.

### Downregulation of NE level impairs LTP in hippocampal CA1 region of adult rats

**Figure 5:**
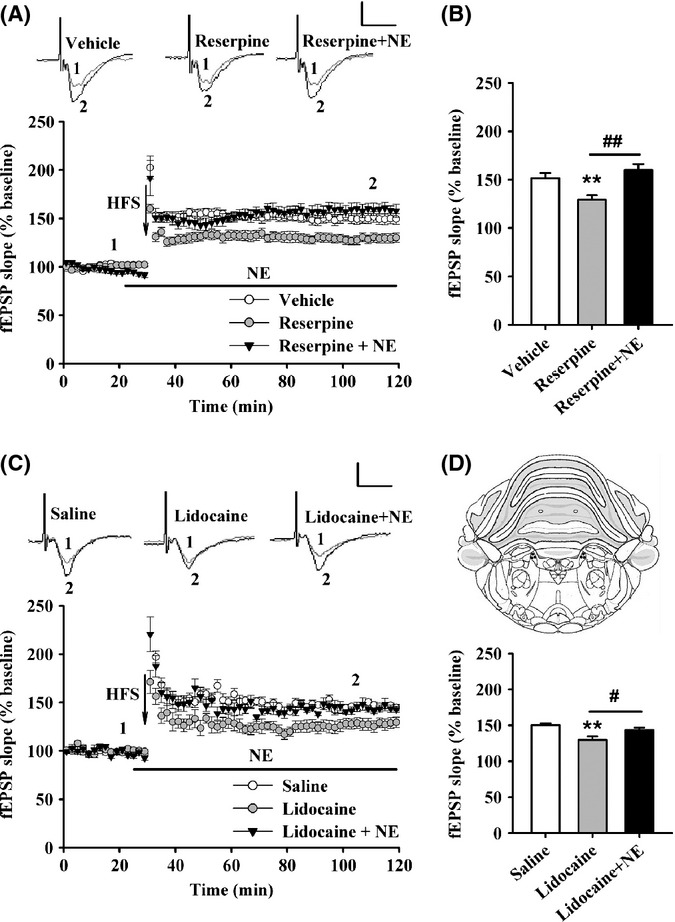
Downregulation of norepinephrine (NE) impairs long-term potentiation (LTP) in hippocampal CA1 region of adult rats. (A) Time course of the field excitatory postsynaptic potential (fEPSP) from vehicle (0.1% DMSO in saline, i.p., *n* = 9 slices from five rats), reserpine (5 mg kg^−1^ in 0.1% DMSO, i.p., *n* = 13 slices from seven rats), and reserpine + 10 μmNE-treated (*n* = 8 slices from four rats) adult rats. (B) The histogram showing the level of LTP at 90 min after high-frequency stimulation (HFS) in vehicle, reserpine, and reserpine + NE-treated adult rats. **P < 0.01 vs. vehicle, ^##^*P* < 0.01 vs. reserpine. (C) Time course of the fEPSP from saline (*n* = 12 slices from seven rats), lidocaine (*n* = 9 slices from five rats), and lidocaine + 10 μmNE-treated (*n* = 8 slices from five rats) adult rats. (D) The histogram showing the level of LTP 90 min after HFS in saline, lidocaine, and lidocaine + NE-treated adult rats. **P < 0.01 vs. saline, ^#^*P* < 0.05 vs. lidocaine. Inset: Representative schematic drawings of cannulae tip positions (black dots) in the locus coeruleus (LC). A coronal viewed at position 3.4 mm posterior to lambda adapted from the atlas of Paxinos & Watson (2007) The Rat Brain Sixth edition. Elsevier Inc. Calibration: 1 mV, 10 ms.

While treatment with NE rescued LTP impairment in aged rats, we supposed whether the downregulation of NE can lead to an impairment of LTP in adult rats. To test this assumption, adult rats were administrated with reserpine (5 mg kg^−1^, i.p.) to interfere with vesicular storage of monoamine neurotransmitters (Zhu *et al*., 2007), or equal volume of vehicle 24 h prior to the preparation of brain slices. We found that reserpine impaired LTP, while acute treatment of NE (10 μm) for 10 min rescued this LTP impairment in adult rats (vehicle: 151.35 + 5.50%, reserpine: 129.26 + 4.66%, reserpine + NE: 159.98 + 6.16%; *F*_2,27_ = 9.154, *P* = 0.001; one-factor ANOVA with the treatment as the factor; Fig.5A,B). LC is the main source of noradrenergic innervations to the hippocampus. To exclude the effects of other monoamine neurotransmitters (dopamine or serotonin), we temporarily suppressed the activity of LC by intra-LC injection of lidocaine (Lashgari *et al*., 2008). Forty-five minutes after the injection, it was found that lidocaine suppressed hippocampal LTP, and this suppression was reversed by acute treatment of NE (saline: 150.30 + 2.80%, lidocaine: 129.56 + 5.27%, lidocaine + NE: 143.45 + 3.56%; *F*_2,26_ = 7.760, *P* = 0.002; one-factor ANOVA with the treatment as the factor; Fig.5C,D). These results suggest that the decrease in NE level leads to LTP impairment in the hippocampal CA1 region of adult rats.

### Acute administration of NE or desipramine rescues aging-related deficits emotional memory

**Figure 6:**
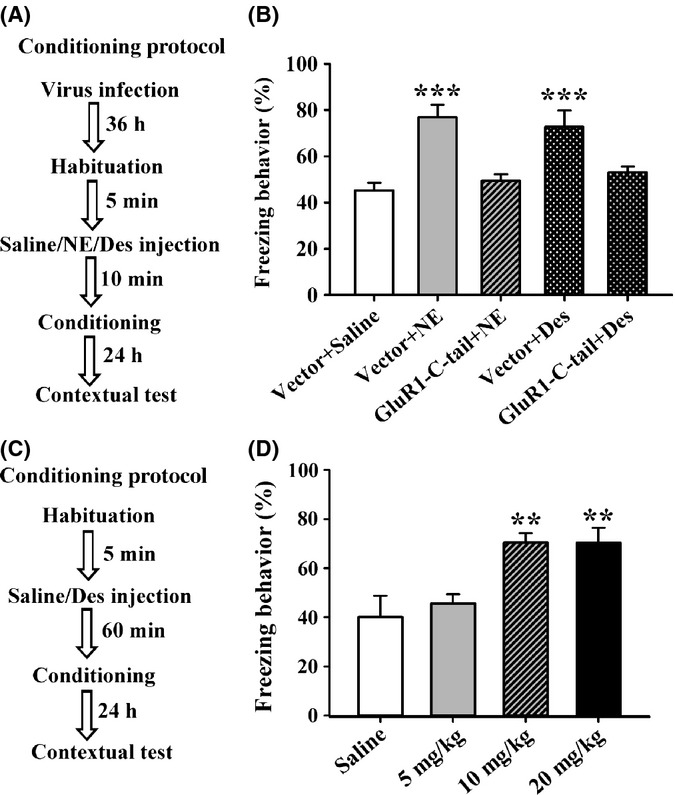
Acute administration of norepinephrine (NE) or desipramine rescues the deficit of contextual fear conditioning memory of aged rats. (A) The contextual fear conditioning procedure. (B) Compared with control (*n* = 7), the freezing behavior was significantly increased in both NE (1 nmol, *n* = 8) and Des (1 μg, *n* = 9)-treated aged rats with control vector infection at 24 h after conditioning training. ***P < 0.001 vs. control. (C) The contextual fear conditioning procedure. (D) The percentage of freezing behavior was significantly increased in Des (10 or 20 mg kg^−1^, i.p.)-treated (*n* = 8) aged rats at 24 h after conditioning training. **P < 0.01 vs. saline.

Considering that acute treatment of NE recovered the LTP *in vitro*, we hypothesized that increasing NE in the brain is able to rescue aging-related emotional memory deficit *in vivo*. It is well known that desipramine improves mood by elevating the level of NE in CNS (Dell'Osso *et al*., 2011). We then supposed that administration of desipramine can rescue the aging-related deficit of emotional memory. To test the hypothesis, we carried out fear conditioning protocol (Fig.6A). As shown in Fig.6B, 24 h after training, aged rats that injected bilaterally with vector adenovirus and NE (1 nmol) exhibited increased freezing behavior (76.92 + 5.36%) when compared with vector adenovirus and saline-treated group (45.20 + 3.27%). Overexpression of GluR1-C-tail significantly reduced the freezing behavior in NE-treated rats (49.43 + 2.79%). Furthermore, bilateral injection of desipramine (1 μg) increased the freezing behavior in aged rats (72.64 + 7.21%), and this effect was inhibited by GluR1-C-tail overexpression (53.09 + 2.45%; *F*_4,33_ = 8.237, *P* < 0.001; one-factor ANOVA with the treatment as the factor). One-factor ANOVA with the treatment as the factor showed that no differences were observed among the groups in baseline behavior before training (*F*_4,33_ = 1.149, NS), freezing behavior during the training of fear conditioning (*F*_4,33_ = 0.933, NS), the pain thresholds (*F*_4,33_ = 0.882, NS for flinching; *F*_4,33_ = 0.628, NS for jumping) or the open-field test (*F*_4,31_ = 0.921, NS for locomotive activity; *F*_4,31_ = 0.489, NS for central duration; Fig. S5). Collectively, these results suggest that both desipramine and NE rescue the deficit of emotional memory in aged rats.

Desipramine is generally administered via i.p. injection to experimental animals. Therefore, we tested whether systemic administration of desipramine can rescue aging-related deficit of emotional memory in aged rats. As shown in Fig.6C, the result by one-factor ANOVA with the treatment as the factor indicated that both 10 mg kg^−1^ (70.43 + 3.78%) and 20 (70.31 + 6.16%) mg kg^−1^ desipramine increased the freezing behavior of aged rats (*F*_3,28_ = 7.853, *P* = 0.001), while 5 mg kg^−1^ desipramine had no effect (Fig.6D), suggesting that desipramine exerts the action in a dose-dependent manner. One-factor ANOVA with the treatment as the factor showed that no differences were observed between the groups in baseline behavior before training (*F*_3,28_ = 0.991, NS), freezing behavior during the training of fear conditioning (*F*_3,28_ = 0.580, NS), the pain thresholds (*F*_3,28_ = 0.678, NS for flinching; *F*_3,28_ = 0.336, NS for jumping) or the open-field test (*F*_3,27_ = 1.074, NS for locomotive activity; *F*_3,27_ = 0.528, NS for central duration; Fig. S6). Taken together, these results suggest that systemic administration of desipramine also rescues aging-related deficit of emotional memory, without changing the levels of innate fear and anxiety, and the effects were not likely attributed to a sensory or motor deficit.

## Discussion

The present study demonstrated for the first time that NE played a key role in the formation of hippocampal synaptic plasticity and the emotional memory in aged rats. The aging-related deficit in emotional memory was accompanied by the decrease in NE level, which hindered the phosphorylation of Ser831 and Ser845 sites of GluR1, and thus reduced the surface expression of GluR1 and impaired the LTP in hippocampus during fear conditioning. On the other side, treatment with NE increased the surface expression of GluR1 and attenuated the impairment of LTP in hippocampal CA1 region of aged rats. Furthermore, acute administration of NE repaired the aging-related deficit of surface expression of GluR1 and emotional memory in aged rats. This effect was also mimicked by systemic administration of desipramine. These findings demonstrate that NE and its related signaling pathways play an important role in the development of the impairment of synaptic plasticity and associative fear memory during aging, and desipramine may represent a brilliant drug for the treatment of aging-related deficit of emotional memory.

The fear conditioning test is commonly used to study fear-/emotion-based learning and memory in rats. Recently, Neff *et al*. (2013) reported that aged mice displayed impaired contextual memories, but the impact of age was not significant. Another previous study reports that the emotional and contextual memories were preserved, but acquisition was slightly impaired in aged rats (Bergado *et al*., 2011). In contrast, our present study found that aging impaired the consolidation, but did not affect the baseline behavior or the acquisition of fear memory in contextual fear conditioning. This discrepancy may be due to the different response to fear conditioning training in different species (mouse and rat, even Wistar and SD rat), and the various experimental conditions. For example, the conditioning training parameters in previous study were 0.8 mA, three pairings of foot shocks with randomized intertrial interval. However, the parameters in our study were 0.75 mA, six pairings of foot shocks with 90 s intertrial interval. And the parameters of contextual test are also different, which is 3 min in the present study, but 5 min in previous study.

Although LTP is a type of synaptic plasticity characterized by an increase in synaptic strength and believed to be involved in memory encoding, how complex memories are stored and recalled at the neuronal circuit level is still not fully understood. Apart from LTP, there are other mechanisms for modulation of memory. For example, memory is believed to be ensembles of cells used to store memories. The hippocampal dentate gyrus and CA3 regions are essential for generating contextual memories of fear, and the strength of memory is related to the reactivation that originates in the hippocampal CA3 region (Denny *et al*., 2014). Therefore, while LTP was abolished in aged rats, there were other mechanisms for memory storage, contextual fear conditioning at such a significant level was still seen in the present study.

Norepinephrine is widely considered to be one of the major neurotransmitters during emotional events (Soeter & Kindt, 2011; Amihaesei & Mungiu, 2012). The hippocampus is a key component of the neural circuitry underlying fear responses (Ferreira *et al*., 2013; Opmeer *et al*., 2013; Pristera *et al*., 2013; Soya *et al*., 2013; Swart *et al*., 2013). Previous studies have reported that NE levels increase in the brain during emotional events (Hayley *et al*., 2001), but decrease in dissected hippocampus and cingulate cortex during spatial memory impairment (Birthelmer *et al*., 2003; Collier *et al*., 2004). Thus, microdialysis was used to measure the extracellular concentration of NE in hippocampal CA1 region during fear conditioning in aged rats. Hu and Katsuki found that NE and β-adrenergic stimulation has profound effects on the induction of LTP via phosphorylation of GluR1-831/845 (Katsuki *et al*., 1997; Hu *et al*., 2007). For example, selective loss of NE exacerbates early cognitive dysfunction and synaptic deficits in APP/PS1 mice (Hammerschmidt *et al*., 2013). NE also reduces the threshold for LTP induction and memory formation in adult mice (Hu *et al*., 2007).

Interestingly, our results indicate that NE increases the amplitude, but not reduces the threshold for LTP induction in aged rats. This may be due to the decreased concentration of extracellular NE and reduced surface expression of GluR1 in aged rats. During the LTP induction, a Ca^2+^ influx through the NMDAR leads to two consequences: a phosphorylation of AMPAR and an incorporation of extrasynaptic AMPAR into the postsynaptic membrane. Our previous study showed that the function of NMDAR was impaired during aging (Yang *et al*., 2010). The possible explanation is that the function of NMDAR in aged rats may be not as significantly enhanced as young rats during HFS. Therefore, the phosphorylation of AMPAR may be also defective in aged rats. In our study, it was found that the effect of NE on phosphorylation of AMPAR can overcome the deficiency of NMDAR for LTP induction in aged rats. It has been reported that during MFS, the activity of NMDAR in aged rats is decreased (Katsuki *et al*., 1997). Consistent with this observation, NE alone cannot induce LTP in our study. On the other hand, the function of NMDAR is intact in young rats. During HFS, the phosphorylation of AMPAR may be nearly saturated, and thus, the effect of NE is determined by the function of NMDAR, thereby, NE cannot increase the amplitude of LTP (Katsuki *et al*., 1997; Hu *et al*., 2007). However, during MFS, as the activation of NMDAR is not fully enough to induce LTP in adult rats, NE and activated NMDAR cooperate to trigger LTP (Katsuki *et al*., 1997; Hu *et al*., 2007), just as NE can lower the threshold for induction of LTP in young rats. Meanwhile, the decrease in NE level in the hippocampus of adult rats resulted in slight impairment of LTP. These results suggest that the decrease in NE level may be one of the important alterations, but not the only change in aged rats. Besides, dopamine, the thiol redox status and NMDAR function are also impaired during aging (Yang *et al*., 2010; Kumar & Foster, 2013; Lin *et al*., 2013).

In our study, it is interesting to note that the surface expression of AMPAR was increased, but the basal fEPSP was unchanged after NE treatment in aged rats. This may be attributed to the delivery of AMPARs near, but not at the synaptic membrane by NE stimulation. During the induction of LTP, AMPARs undergo PKA/CaMKII-dependent insertion at perisynaptic sites, where they are initially stabilized by actin polymerization, and then translocate into the synapses to induce the expression of LTP (Yang *et al*., 2008; Henley & Wilkinson, 2013).

Long-term potentiation has been proposed to be involved in memory encoding since it was found in 1973 (Bliss & Gardner-Medwin, 1973). However, it is difficult to verify the direct relationship between LTP and memory (Stevens, 1998). It has been a great challenge to demonstrate the causal link between them in neuroscience for the past 41 years. Recently, Nabavi *et al*. (2014) have engineered reactivation of a memory using LTP, supporting a causal link between LTP and memory. Thus, it is reasonable to deduce that NE regulated the trafficking of AMPAR through β receptor, then enhanced the amplitude of LTP, and finally improved the memory of contextual fear conditioning in aged rats. Further experiments are necessary to verify this relationship in the next experiment.

Desipramine has been used widely as an inhibitor of NE reuptake. Given that appropriate extracellular concentration of NE is required for rescuing aging-related deficit of LTP, we hypothesized that desipramine should be effective in repairing the aging-related memory deficit. We found that the acute administration of desipramine was successful in improving emotional memory in aged rats, suggesting that desipramine may have therapeutic effect on the aging-related deficit of emotional memory.

In conclusion, our results demonstrate for the first time that the decreased level of NE in the hippocampus is responsible for aging-related deficit of emotional memory. Acute administration of NE or desipramine rescues the deficit via regulating the surface expression of GluR1 in aged rats. The antidepressant drug desipramine may represent an effective medication for the treatment of aging-related emotional memory deficit.

## Experimental procedures

### Electrophysiological recording

Transverse hippocampal slices (350 μm) from adult or aged rats were obtained as our previous studies with some modifications (Yang *et al*., 2010; Luo *et al*., 2014). Briefly, the hippocampal slices were cut with a vibratome (VT 1000S; Leica, Wetzlar, Germany), after recovery for at least 1 h at room temperature (25 ± 1 °C) in artificial cerebrospinal fluid (ACSF). Then, an individual slice was transferred into a submerged recording chamber and continuously superfused with ACSF at room temperature (25 ± 1 °C) at a rate of 3–4 mL min^−1^. To evoke LTP, HFS was induced after recording fEPSPs at least 15 min. HFS consisted of three trains of 100 pulses at 100 Hz separated by 30 s and delivered at test intensity. Middle frequency stimulation (MFS) consisted of a total of 900 pulses delivered at 10 Hz for 90 s. Test with single pulse was then resumed for 90 min to determine the level of stable LTP. LTP data were acquired without parallel recordings from a nontetanized control pathway.

### Statistical analysis

All analyses were performed using spss 18.0 software (SPSS Inc., Chicago,Illinois, USA), and data are presented as mean ± SEM. To compare NE levels at the time points relative to fear conditioning or administration of epinephrine between groups, a multifactorial ANOVA was used after repeated measure ANOVA. The expression of GluR1 and GluR2 after fear conditioning training between groups was statistically evaluated using multifactorial ANOVA. Other results from fear conditioning, Western blot, microdialysis, and the level of stable LTP were statistically evaluated using one-way ANOVA unless mentioned. *Post hoc* tests were performed using least significant difference test. *P *< 0.05 was considered statistically significant.

Chemicals, Animals, Surface protein cross-linking with BS^3^, surgery and microdialysis and injection, Western blotting, adenovirus infection and overexpression, HPLC analysis of NE, Fear conditioning tasks, measurement of pain threshold and open-field test were described in Appendix S1.

## Supporting Information

**Appendix S1** Supplementary Materials and Methods. **Fig. S1** No differences in baseline behavior, freezing behavior, pain thresholds and the open field test are observed between adult and aged rats except activity suppression ratio. **Fig. S2** The field responses were stable at baseline in the SC-CA1 pathway. **Fig. S3** The short-term memory is impaired in aged rats and the extracellular concentration of NE is decreased in the hippocampus of aged rats, with no changes in the surface expression of GluR2. **Fig. S4** The effects of NE on GluR1, PKC, ERK and CREB in the hippocampus of aged rats. **Fig. S5** Adenovirus-mediated GluR1 overexpression has no effect on the baseline behavior, freezing behavior during the training of fear conditioning, the pain thresholds or the open-field test. **Fig. S6** Acute administration of desipramine does not affect the performance in the baseline behavior, freezing behavior during the training of fear conditioning, the pain thresholds or the open-field test.
