# Supplementary material for: Reversal of aging-related emotional memory deficits by norepinephrine via regulating the stability of surface AMPA receptors
Source: Aging Cell. 2015 Jan 7;14(2):170–9. doi: 10.1111/acel.12282 (PMC4364829; doi:10.1111/acel.12282)
Supplement: Supplementary file 1 [file acel0014-0170-sd1.doc]

**Supplemental Information**

**1. Supplementary Materials and Methods**

**Chemicals**

(±)-Norepinephrine (+)-bitartrate salt (NE), (±)-propranolol hydrochloride (Pro), phentolamine hydrochloride (Phen), (−)-epinephrine, reserpine, lidocaine hydrochloride and desipramine hydrochloride (Des) were purchased from Sigma (St. Louis, MO, USA). Other general agents were purchased from commercial suppliers. Before application, the stock solution of chemicals was freshly diluted with artificial cerebrospinal fluid (ACSF) containing (in mM) 119 NaCl, 3.5 KCl, 1.3 MgSO_4_, 2.5 CaCl_2_, 1 NaH_2_PO_4_, 26.2 NaHCO_3_, and 11 glucose, pH 7.4. When dimethyl sulfoxide (DMSO; Sigma) was used to prepare solutions and its final concentration was 0.1% or less.

**Animals**

The research was conducted with the Guide for Care and Use of Laboratory Animals as adopted and promulgated by the United States National Institutes of Health. The use of animals for experimental procedures was approved by the Animal Welfare Committee of Huazhong University of Science & Technology. The experiments were conducted in 4- to 5-month-old and 20- to 24-month-old male Sprague-Dawley rats. Rats were maintained on a controlled light-dark cycle at a constant temperature (22 ± 2 °C) with ad libitum access to food and water.

**Fear conditioning tasks**

The procedures of fear conditioning were processed according to our previous studies with some modifications . Briefly, on day 1, rats were habituated in the training chamber (30×25×30 cm^3^) for 5 min before fear conditioning (habituation). Then, the rats were presented with 6 foot shock pairings (1 s). The intertribal-interval (ITI) was 90 s and the shock intensity was 0.75 mA (conditioning). The chamber was cleaned thoroughly after each trial. After training, the rats were returned to their home cages. To assess contextual fear memory, rats were placed into the same conditioning chamber 24 h later, and observed for 3 min (test). Contextual memory was assessed by measurement of time spent freezing. Conditioned freezing was defined as immobility, except for respiratory movements. The total freezing time in the test period was represented as a percentage. Activity suppression ratio was calculated as follows: Activity during the context test / (activity during the context test + activity during the baseline), where “context test” refers to the test that is given 1 day after context fear conditioning and “baseline” refers to the time period before the onset of the first shock during the day of fear conditioning. 
**Surface protein cross-linking with BS^3^**

The cross-linking of receptor in plasma membrane was performed according to our previous report with slight modifications . The brain was removed quickly after the rat was decapitated and coronal hippocampal slices (350 μm) were made with a vibratome (VT 1000S; Leica) in ice-cold ACSF containing (in mM) 119 NaCl, 3.5 KCl, 1.3 MgSO_4_, 2.5 CaCl_2_, 1 NaH_2_PO_4_, 26.2 NaHCO_3_, and 11 glucose, pH 7.4. After cutting, the hippocampal slices were retained for recovery in oxygenated ACSF at 25 ± 1 °C for 1 h. After treatments, the hippocampal slices were dissected and added to eppendorf tubes containing ice-cold ACSF, which was spiked with 1 mM bis (sulfosuccinymidal) suberate (BS^3^; Thermo Scientific, Rockford, IL, USA) immediately after addition of tissue. Then the tissue was cross-linked for 30 min at 4 °C with gentle agitation and this reaction was terminated by quenching with 100 mM glycine (15 min at 4 °C). Then the tissue was pelleted by brief centrifugation, re-suspended in ice-cold lysis buffer containing protease and phosphatase inhibitors (in mM) (50 Tris-base, 100 NaCl, 10 EDTA, 20 NaF, 1 PMSF, 3 Na_3_VO_4_, 1% NP-40, protease inhibitor mixture, pH 7.4), homogenized rapidly by sonicating for 10 s, and centrifuged (12000 g for 15 min at 4 °C). The supernatant fraction was separated and stored at -80 °C before being used for Western blotting.

**Surgery and microdialysis and injection**

The rats were anesthetized with sodium pentobarbital (60 mg/kg) via intraperitoneal injection (i.p.) and placed in stereotaxic apparatus.

For microdialysis, a guide cannula was unilaterally stereotaxically implanted into the dorsal hippocampus of CA1 region (As the brain of aged rat is larger than adult, AP-3.0 mm, ML-2.2 mm, DV-1.5 mm for adult; AP-3.2 mm, ML-2.3 mm, DV-1.7 mm for aged from the bregma and dural surface). The cannulae were fixed to the skull with the aid of jeweler screws and dental acrylic resin skull and cranioplastic dental cement. After implantation of the cannula, rats were habituated to cages for at least 5 d before further experiments. After this recovery period, a microdialysis probe (with active region 2.0 mm in length) was inserted through the guide cannula and microdialysis sample collection was started. ACSF was perfused through the dialysis fiber at the rate of 2.0 μl/min by a microinfusion pump. After at least 30 min washout period, the perfusate fractions were collected every 20 min. After fear conditioning, the perfusate fractions were continued to be collected every 20 min.

For intra-CA1 injection, two stainless steel cannulas with 12 mm length and 0.6 mm outside diameter were bilaterally implanted into the hippocampus CA1 region (AP-3.0 mm, ML-2.2 mm, DV-2.5 mm for adult; AP-3.2 mm, ML-2.3 mm, DV-2.7 mm for aged). To maintain patency, a stylus 0.5 mm longer than the guide cannula was inserted into the guide. After removal of the stylus from cannula, the drugs were injected into the CA1 with a microsyringe (5 μL) connected by a PE-10 polyethylene tubing (10 cm) to a needle (0.3 mm in outside diameter, 1 mm longer than guide cannula), which was introduced into the brain region through the cannula fixed to the head of rat. The injection volume was set to 1 μL within a period of 5 min. The needle was withdrawn over a course of 10 min.

For intra-LC injection, two stainless steel cannulas with 15 mm length and 0.6 mm outside diameter were implanted into the LC region (AP-3.4 mm from lambda, ML±1.2 mm, DV-5.8 mm for adult). Neural activity in the LC was temporarily inactivated by microinjection of 0.5 μL of 4% lidocaine solution within a period of 2 min .

**Western blotting**

Protein samples of hippocampus from experimental animals after treatments were prepared for western blot analysis. The procedures were processed according to our previous protocol with some modifications . In brief, hippocampal tissue from each rat was homogenized with 80 μL of ice-cold lysis buffer that had the same composition as mentioned above. Protein concentrations were examined by Coomassie blue protein-binding assay (Nanjing Jiancheng Institute of Biological Engineering, Nanjing, China). Then, 30 or 50 μg of protein samples were separated by 10% or 6% SDS-polyacrylamide gel and then transferred to nitrocellulose membrane. After blocking with 5% BSA (bovine serum albumin) in Tris-buffered saline containing 0.1% Tween-20 (TBST) for 1 h at room temperature, the transferred membranes were incubated overnight at 4 °C with primary antibodies: anti-β-actin (1:3000) (Upstate Biotechnology, Lake Placid, NY, USA); anti-phospho-CaMKII (1:400) and anti-CaMKII (1:500) (Cell Signaling, San Francisco, CA, USA); anti-phospho-ERK1/2 (1:500) and anti-ERK1/2 (1:500) (Santa Cruz Biotechnology, Santa Cruz, CA, USA); anti-phospho-PKA C (Thr197) (1:800) and anti- PKA C-α (1:1000) (Cell Signaling); anti-phospho-PKC (1:800) and anti-PKC (1:1000) (Cell Signaling); anti-phospho-CREB (1:500) and anti-CREB (1:1000) (Cell Signaling); anti-phospho-GluR1 (Ser845) (1:400), anti-phospho-GluR1 (Ser831) (1:400), anti-GluR1 (1:500) and anti-GluR2 (1:500) (Cell Signaling). Following three washes with TBST, membranes were then incubated with horseradish peroxidase (HRP)-conjugated secondary antibodies (1:3000) (Sigma, St. Louis, MO, USA) in TBST with 3% BSA for 1 h at room temperature. After repeated washes, membranes were reacted with enhanced chemiluminescence reagents (Super Signal West Pico; Pierce Chemical Co., Rockford, IL, USA). Images were scanned and captured with Micro Chemi (DNR Bio-imaging systems, Jerusalem, Israel) and the optical densities of the detected bands were quantified using Scion Image software (Fredrick, MD, USA). The loading volumes of the samples were determined based on the protein concentrations, and the optical densities of internal reference (β-actin) were uniform among sample lanes. All assays were performed at least three times. Results are presented as percentage of control after normalization.

**Adenovirus infection and overexpression**

The gene sequence of GluR1 carboxyl terminus (CGAGTTCTGCTACAAATCCCGTAGCGAGTCGAAGCGGATGAAGGGTTTCTGTTTGATCCCACAGCAATCCATCAATGAAGCCATACGGACATCGACCCTCCCCCGGAACAGTGGGGCAGGAGCCAGCGGAGGAGGCGGCAGTGGAGAGAATGGCCGGGTGGTCAGCCAGGACTTCCCCAAGTCCATGCAATCCATTCCCTGCATGAGTCACAGTTCAGGGATGCCCTTGGGAGCCACAGGATTGTAA) was obtained as previously described . The recombinant adenoviruses encoding rat GluR1-C-terminal tail (CV049), which contains a Ubi-driven enhanced green fluorescent protein (EGFP) reporter and cloning restriction sites (NheI/EcoRI) to allow introduction of GluR1 carboxyl terminus, and control vector adenovirus (GV137) were constructed, amplified and purified to be 1×10^11^ PFU/mL. Adenovirus was purchased from Shanghai Genechem Co., Ltd. (Shanghai, China). 2.0 μl of adenovirus suspension was injected in each hemisphere at a rate of 0.2 μl/min. Rats were subjected to experiments 36 h after adenovirus injection.

**High-performance liquid chromatography (HPLC) analysis of norepinephrine**

Norepinephrine was measured by reverse-phase-HPLC (2.1 × 150 mm, C18, 3 μm, Antec ALF-215) coupled to electrochemical detection (Antec technologies, the Netherlands) (HPLC-ECD), The mobile phase was 37.5 mM sodium dihydrogen phosphate, 0.85 mM 1-octanesulfonic acid sodium salt, 100 μL triethylamine (TEA), 12.5 μM EDTANa_2_, 10% acetonitrile (V/V), set to pH 3.0. The flow rate of the pump was 0.15 ml/min and the working electode was +0.7 V versus Ag/AgCl reference electrode. The column temperature was kept at 30 °C and the injection volume was 10 μL. For the linear calibration plot test, a series of NE solutions with the concentration in a range from 50 pM to 1000 pM were chosen and injected with at least once in the HPLC-ECD, this device gave a similar response, producing a stable baseline. In these experiments, all injections were made using an Antec AS110 autosampler. The results showed that peak areas for NE were a linear function of concentration with a correlation coefficient of 0.9950. Data was analyzed by a regression analysis test.

**Measurement of pain threshold**

Rats were placed individually into the conditioning chamber with electric grid. After 3 min of retention, electric foot shocks (1 s) were applied starting with an intensity of 0.1 mA. The intensity was increased gradually by 0.1 mA (with pauses of 30 s between successive stimuli) until the animal showed the first signs of pain (flinching or jumping), and the corresponding intensity was taken as the pain threshold .

**Open field test**

Rats were placed individually into the activity chamber (40×40×40 cm^3^) and monitored by a video motility system. The 20×20 middle part of the activity chamber was defined as central part. The spontaneous motor activity including total distance and the anxiety-like behavior activity including total spending time in the central part during 30 min intervals were monitored and assessed .

**2. Supplementary Figures**


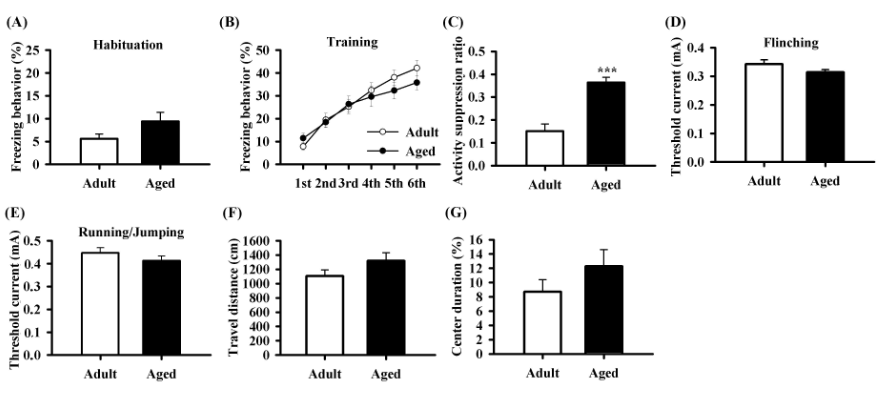


*Fig. S1. No differences in baseline behavior, freezing behavior, pain thresholds and the open field test are observed between adult and aged rats except activity suppression ratio.* (A) Adult (n=10) and aged (n=7) rats exhibited similar freezing behavior in conditioning chamber before conditioning training and (B) during conditioning training. (C) Aged rats exhibited a higher activity suppression ratio (D) Thresholds of shock intensities for flinching. (E) Thresholds of shock intensities for running/jumping. No difference was detected between adult (n=10) and aged (n=8) rats. (F) Total distance moved and (G) the percentage of time spent in central part during 30 min intervals in open field test. No difference was observed between adult (n=10) and aged (n=10) rats.


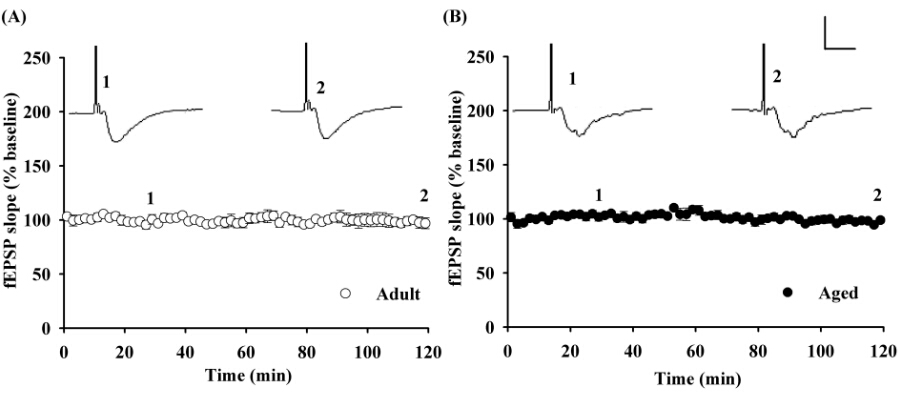


*Fig. S2. The field responses were stable at baseline in the SC-CA1 pathway.* (A) Adult (n=5) and (B) aged (n=6) rats. Calibration: 1 mV, 10 ms.


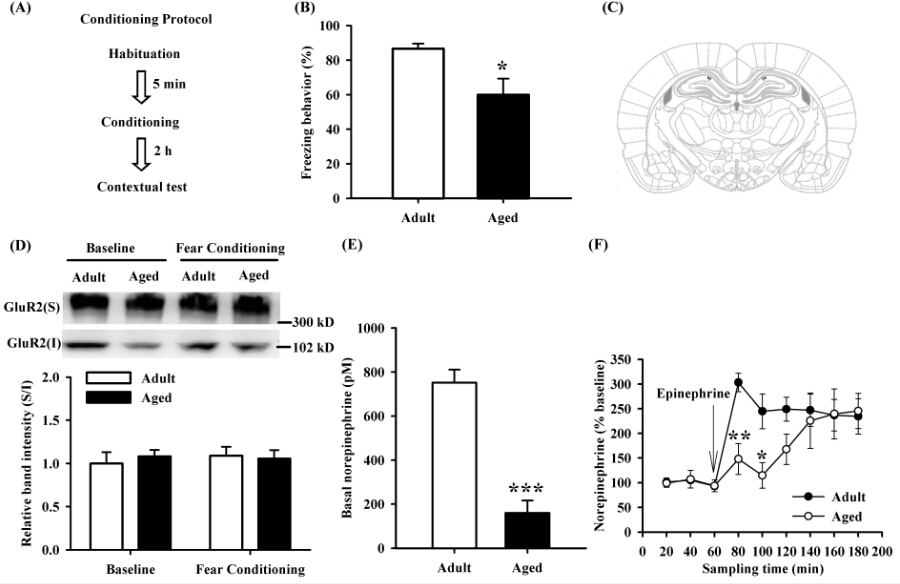


*Fig. S3. The short-term memory is impaired in aged rats and the extracellular concentration of NE is decreased in the hippocampus of aged rats, with no changes in the surface expression of GluR2.* (A) The contextual fear conditioning procedure. (B) The freezing behavior was significantly reduced in aged rats (n=9) 2 h after conditioning training compared with adult rats (n=10). *P<0.05 vs adult. (C) Representative schematic drawings of microdialysis probe positions (black dots) in the dorsal hippocampus of CA1. A coronal viewed at position 3.0 mm posterior to bregma adapted from the atlas of Paxinos & Watson (2007). (D) Representative images of western blotting (upper). Protein extracts from the hippocampus were analyzed by western blotting at baseline and 1 h after fear conditioning. There were no difference in the surface expression of GluR2 between aged rats (n=6) and adult rats (n=6). (E) Aged rats (n=7) exhibited a lower concentration of extracellular NE in CA1 compared with adult rats (n=7). (F) Microdialysis of extracellular NE concentration in dorsal hippocampus of CA1 region before and after administration of epinephrine (0.5 mg/kg, i.p.). The level of extracellular NE in hippocampus of aged rats (n=6) was lower than that of adult rats (n=7) at 20 min and 40 min after epinephrine injection. *P<0.05, **P<0.01, ***P<0.001 vs adult.


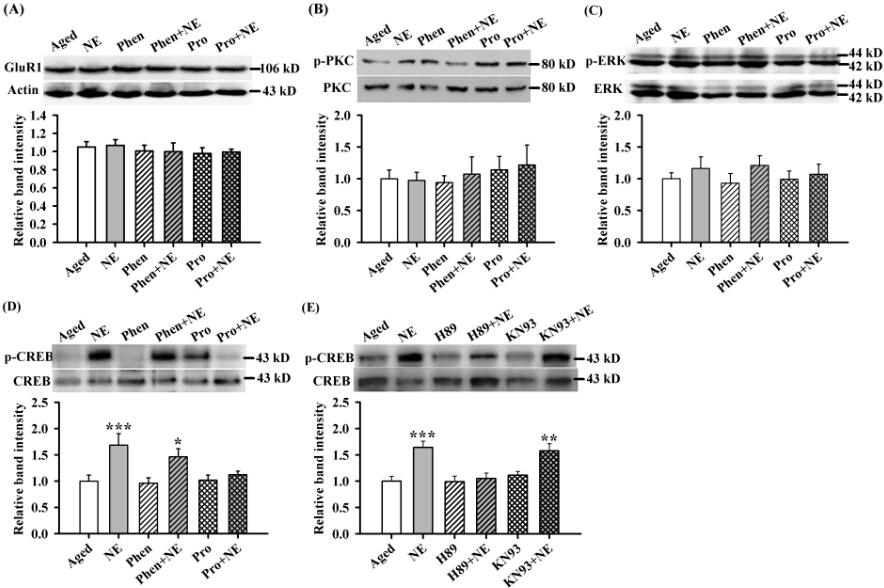


*Fig. S4. The effects of NE on GluR1, PKC, ERK and CREB in the hippocampus of aged rats.* (A) The total expression of GluR1 was unaffected by NE in aged rats (n=6). (B) PKC signaling was unaffected by NE in aged rats (n=6). (C) ERK signaling was unaffected by NE in aged rats (n=6). (D) Representative images of western blotting (upper). The histogram showing that the phosphorylation level of CREB was enhanced by NE, which was prevented by pretreatment with Pro, but not Phen (n=6). *P<0.05, ***P<0.001 vs aged rats. (E) Representative images of western blotting (upper). The histogram showing that the phosphorylation level of CREB was enhanced by NE, and this response was inhibited by pretreatment with H89, but not KN93 (n=6). **P<0.01, ***P<0.01 vs aged rats.


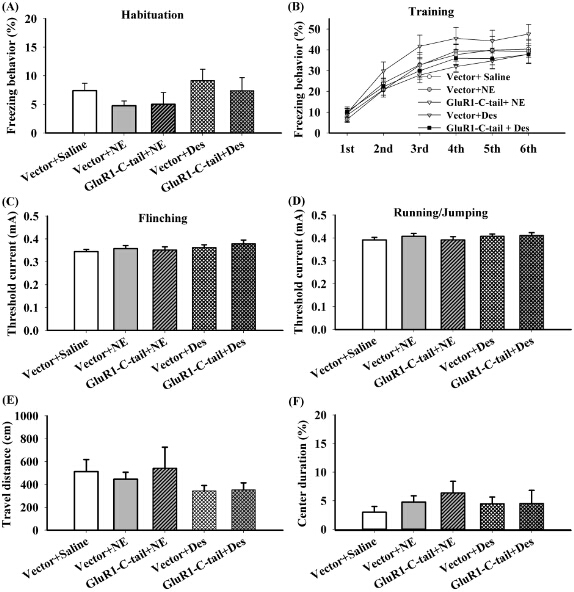


*Fig.S5. Adenovirus-mediated GluR1 overexpression has no effect on the baseline behavior, freezing behavior during the training of fear conditioning, the pain thresholds or the open-field test.* (A) There were no differences in the freezing behavior before conditioning training and (B) during conditioning training between groups. (C) Thresholds of shock intensities for flinching and (D) running/jumping are shown. No difference was detected between groups. (E) Total distance moved and (F) the percentage of time spent in central part during 30 min intervals in open field test. No difference was observed between groups.


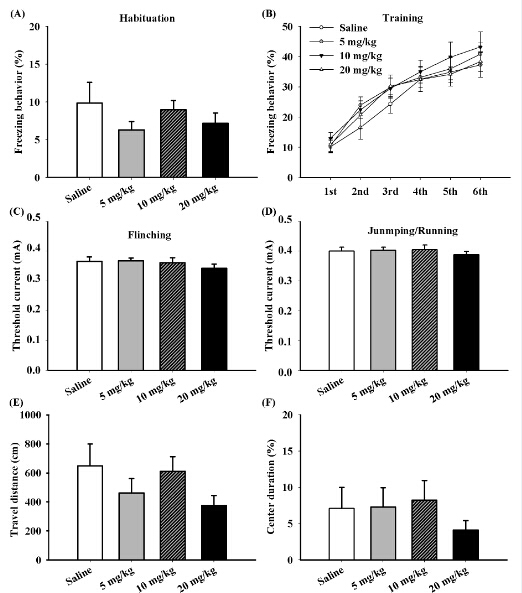


*Fig.S6. Acute administration of desipramine does not affect the performance in the baseline behavior, freezing behavior during the training of fear conditioning, the pain thresholds or the open-field test.* (A) There were no differences in freezing behavior before conditioning training and (B) during conditioning training between groups. (C) Thresholds of shock intensities for flinching and (D) running/jumping are shown. No difference was detected between groups. (E) Total distance moved and (F) the percentage of time spent in central part during 30 min intervals in open field test. No difference was observed between groups.

**References**

Fabris G, Steiner AA, Anselmo-Franci JA , Branco LG (2000). Role of nitric oxide in rat locus coeruleus in hypoxia-induced hyperventilation and hypothermia. *Neuroreport*. **11**, 2991-2995.

Lashgari R, Khakpour-Taleghani B, Motamedi F , Shahidi S (2008). Effects of reversible inactivation of locus coeruleus on long-term potentiation in perforant path-DG synapses in rats. *Neurobiol Learn Mem*. **90**, 309-316.

Li YK, Wang F, Wang W, Luo Y, Wu PF, Xiao JL, Hu ZL, Jin Y, Hu G , Chen JG (2012). Aquaporin-4 deficiency impairs synaptic plasticity and associative fear memory in the lateral amygdala: involvement of downregulation of glutamate transporter-1 expression. *Neuropsychopharmacology*. **37**, 1867-1878.

Lu HF, Wu PF, Yang YJ, Xiao W, Fan J, Liu J, Li YL, Luo Y, Hu ZL, Jin Y, Wang F , Chen JG (2014). Interactions between N-Ethylmaleimide-Sensitive Factor and GluR2 in the Nucleus Accumbens Contribute to the Expression of Locomotor Sensitization to Cocaine. *J Neurosci*. **34**, 3493-3508.

Shi S, Hayashi Y, Esteban JA , Malinow R (2001). Subunit-specific rules governing AMPA receptor trafficking to synapses in hippocampal pyramidal neurons. *Cell*. **105**, 331-343.

Wang W, Wang F, Yang YJ, Hu ZL, Long LH, Fu H, Xie N , Chen JG (2011). The flavonoid baicalein promotes NMDA receptor-dependent long-term potentiation and enhances memory. *Br J Pharmacol*. **162**, 1364-1379.

Yang J, Li MX, Luo Y, Chen T, Liu J, Fang P, Jiang B, Hu ZL, Jin Y, Chen JG , Wang F (2013). Chronic ceftriaxone treatment rescues hippocampal memory deficit in AQP4 knockout mice via activation of GLT-1. *Neuropharmacology*. **75C**, 213-222.
